# Dissemination of a Highly Virulent Pathogen: Tracking The Early Events That Define Infection

**DOI:** 10.1371/journal.ppat.1004587

**Published:** 2015-01-22

**Authors:** Rodrigo J. Gonzalez, M. Chelsea Lane, Nikki J. Wagner, Eric H. Weening, Virginia L. Miller

**Affiliations:** 1 Department of Microbiology and Immunology, University of North Carolina at Chapel Hill, Chapel Hill, North Carolina, United States of America; 2 Department of Genetics, University of North Carolina at Chapel Hill, Chapel Hill, North Carolina, United States of America; Duke University, UNITED STATES

## Abstract

The series of events that occurs immediately after pathogen entrance into the body is largely speculative. Key aspects of these events are pathogen dissemination and pathogen interactions with the immune response as the invader moves into deeper tissues. We sought to define major events that occur early during infection of a highly virulent pathogen. To this end, we tracked early dissemination of *Yersinia pestis*, a highly pathogenic bacterium that causes bubonic plague in mammals. Specifically, we addressed two fundamental questions: (1) do the bacteria encounter barriers in disseminating to draining lymph nodes (LN), and (2) what mechanism does this nonmotile bacterium use to reach the LN compartment, as the prevailing model predicts trafficking in association with host cells. Infection was followed through microscopy imaging in addition to assessing bacterial population dynamics during dissemination from the skin. We found and characterized an unexpected bottleneck that severely restricts bacterial dissemination to LNs. The bacteria that do not pass through this bottleneck are confined to the skin, where large numbers of neutrophils arrive and efficiently control bacterial proliferation. Notably, bottleneck formation is route dependent, as it is abrogated after subcutaneous inoculation. Using a combination of approaches, including microscopy imaging, we tested the prevailing model of bacterial dissemination from the skin into LNs and found no evidence of involvement of migrating phagocytes in dissemination. Thus, early stages of infection are defined by a bottleneck that restricts bacterial dissemination and by neutrophil-dependent control of bacterial proliferation in the skin. Furthermore, and as opposed to current models, our data indicate an intracellular stage is not required by *Y. pestis* to disseminate from the skin to draining LNs. Because our findings address events that occur during early encounters of pathogen with the immune response, this work can inform efforts to prevent or control infection.

## Author Summary

The earliest stage of any infection takes place when a pathogen enters the body (inoculation) at an initial site of contact. From this point, the pathogen can spread into deeper tissues where the pathogen itself and the immune responses against it cause disease. Very little is known about the events that follow inoculation and how pathogens move from the initial site of contact into deeper tissues. A better understanding of this process can potentially result in strategies to control or prevent disease. We studied the highly infectious bacterium that causes bubonic plague (*Yersinia pestis*) and how it spreads inside the body, from the skin into lymph nodes. We found that movement from the skin is highly restricted as only a small fraction of the bacteria that are deposited into this tissue are found in lymph nodes. While it is currently thought that *Y. pestis* spreads from the skin inside trafficking cells of the innate immune response, our work suggests these cells are not required for the bacteria to move into lymph nodes. Our findings can influence vaccine development efforts as these strategies are based on the study of early pathogen interactions with cells of the immune response.

## Introduction

Dissemination is key for a pathogen to reach sites where the environment favors survival or the probability of being transmitted to other hosts is higher. As the pathogen invades new tissues, however, the host responds by eliciting immune responses in an effort to eliminate infection. These interactions define the severity of disease and the outcome of infection. Thus, determining how host and pathogen interact during dissemination is key to understanding disease and to designing strategies to control it.

Particularly relevant questions include what are the events that follow pathogen entrance into the body (i.e. inoculation) and how do these events define dissemination. The answers to these questions are key not only to deepen our understanding of the biology of infection, but, most importantly, to propose strategies that might interrupt pathogen spread in a clinical setting. Remarkably, for the great majority of pathogens, it is still unknown how dissemination into deeper tissues occurs. This is probably because experiments to study host-pathogen interactions *in vivo* can be extremely challenging, especially when using infection models that most closely mimic a natural infection (e.g. relevant route of inoculation, use of virulent strain, etc.). The challenges that are associated with the use of animal models are the main reason why most studies have relied on *in vitro* models to study infection. Notably, most of the current ideas of how host and pathogen interact early during infection derive from these *in vitro* studies.

*Yersinia pestis* is the causative agent of bubonic plague, a severe bacterial disease characterized by aggressive dissemination within the host. This nonmotile bacterium first disseminates from the inoculation site (IS) into the draining lymph node (LN) after inoculation in the skin [1,2]. Colonization of the LN is then followed by bacterial escape into the bloodstream, resulting in septic shock and death [3]; escape into the bloodstream is a necessary step for ultimate transmission of the bacteria to a new host. The ability of *Y. pestis* to efficiently disseminate makes it an unparalleled model to study bacterial dissemination *in vivo* and to understand how a host responds to the threat of severe infection. Successful colonization of the host depends on the expression of bacterial virulence factors (e.g. type III secretion system, pH 6 antigen, F1 antigen) that are upregulated at 37°C and prevent phagocytosis [3–5]. These antiphagocytic factors are predicted to be expressed at low levels during the first hours of infection, a notion that gave rise to the hypothesis that an intracellular stage facilitates trafficking from skin to LN [6,7]. This is partially supported by *in vitro* experiments showing bacterial survival in macrophages [6]. Whether phagocytic cells are required for *Y. pestis* dissemination from the skin into the LN is still unknown.

The goal of this study was to define what events occur immediately after inoculation of *Y. pestis* into the skin and how these events affect bacterial dissemination. Specifically, we sought to define the host-pathogen interactions that occur during dissemination. Most importantly, we were interested in testing whether *Y. pestis* requires phagocytic cells to disseminate from the skin into the draining LN.

## Results

### Visualization of bacteria in the skin, LNs and lymphatic vessels connecting both tissues

*Y. pestis* survives in multiple tissues during infection. This has been shown extensively through experiments where tissues of infected animals are harvested to obtain bacterial burdens. However, with such an approach it is not possible to make observations of bacteria in the context of the different niches the pathogen interacts with in the host as it disseminates. With the aid of confocal microscopy of whole mounts (i.e. not sections), we visualized *Y. pestis* at the major anatomical sites the bacteria travels through during infection. An intradermal (ID) model of infection where ∼200 colony forming units (CFU) are injected in the ear pinna was used to mimic the delivery into the dermis that occurs during a flea bite. We chose this dose as it is highly relevant given that 83.7% of mice inoculated by a flea receive <500 CFU and 93.5% receive <1000 CFU[8]. While ID models of infection previously have been reported [9–11], our model is based on the use of a particularly small volume of injection (2 μL). A small volume reduces the possibility of confounding effects derived from tissue damage caused by larger volumes disrupting the dermis. After ID inoculation with *Y. pestis* expressing *rfp* (RFP-*Y. pestis*) or *gfp* (GFP-*Y. pestis*), whole mounts were fixed and imaged using confocal microscopy. With this approach, we obtained images of bacteria in tissues whose architecture was minimally disrupted without compromising the use of a fully virulent strain of *Y. pestis*.

**Figure 1:**
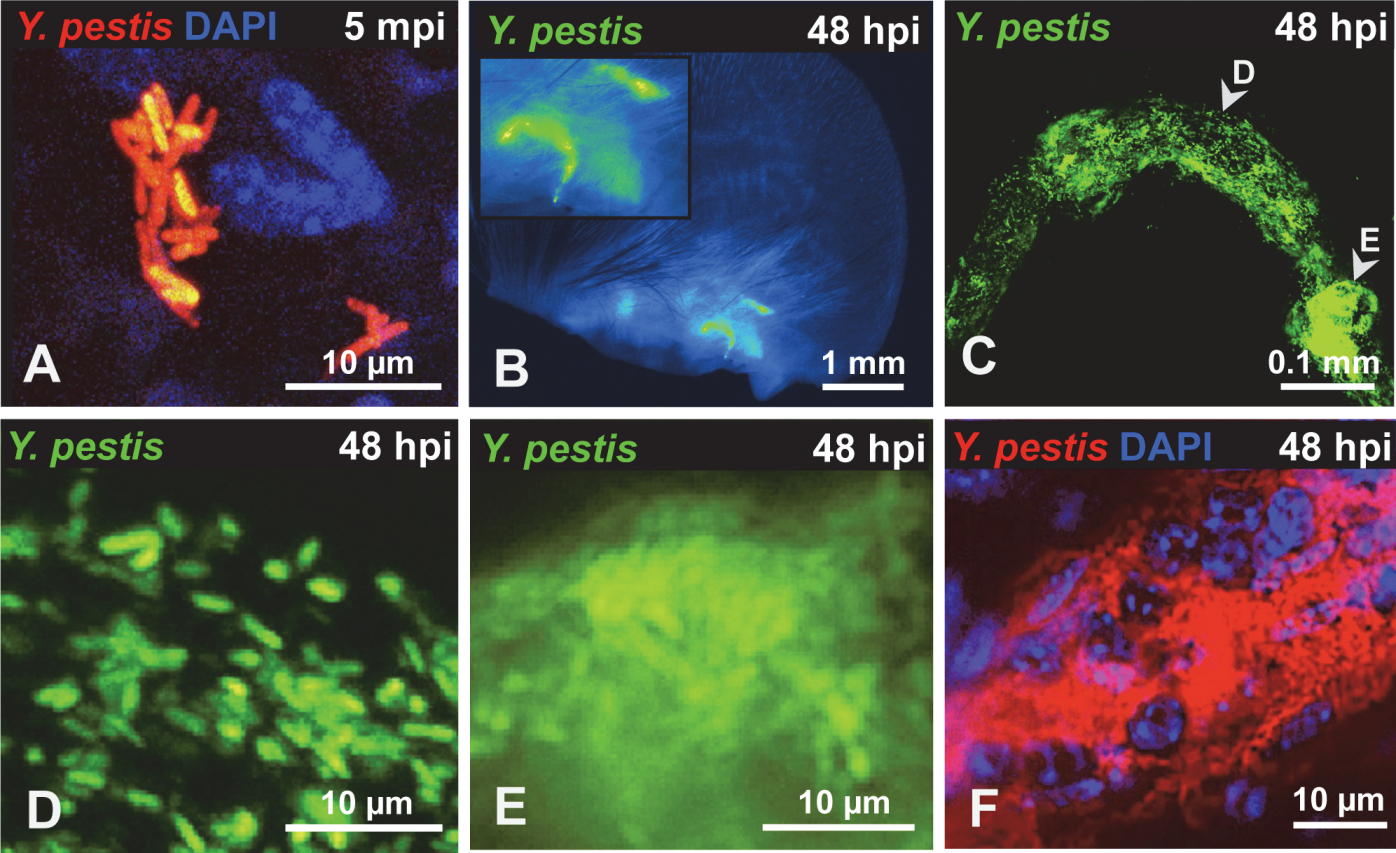
*Y. pestis* in the skin and in afferent lymphatic vessels. (A) RFP-*Y. pestis* in the skin 5 minutes post inoculation (mpi). (B) GFP-*Y. pestis* in tube-like structures at the base of a mouse ear. Signal from the ear tissue derives from autofluorescence and was false-colored blue. (C) GFP-*Y. pestis* in tubes located at the base of the ear (arrowheads indicate regions enlarged in D and E). (D-E) *Y. pestis* inside the tubes. (F) RFP-*Y. pestis* associated with host cells (nuclei stained with DAPI) inside tubes. (B-F) taken at 48 hpi. A, C, and F show maximum intensity projections from z-stacks. Experiments were performed a minimum of 3 times and representative pictures are shown.

Bacteria were localized exclusively at two specific sites in the ear. One was the injection site, defined by the small and transient wheal (bubble) that forms in the skin during inoculation (S1 A-C Fig.). Bacteria at this site were found mostly in large clumps or as small groupings of cells (Fig. 1A). The second site where bacteria were found was at the base of the ear in the shape of tube-like structures (presumably lymphatic vessels), noted at 24 hours post inoculation (hpi). The presence of these tubes was infrequent (approximately 1 in 10 mice) but when seen, signal was so strong that it could be seen easily at lower magnifications (Fig. 1B). Bacteria were distributed unevenly in these tubes and appeared as individual or very tight clumps of bacteria (Fig. 1C, D, and E). Staining with DAPI revealed the presence of host cells in very close proximity to bacteria inside of these tubes (Fig. 1F).

**Figure 2:**
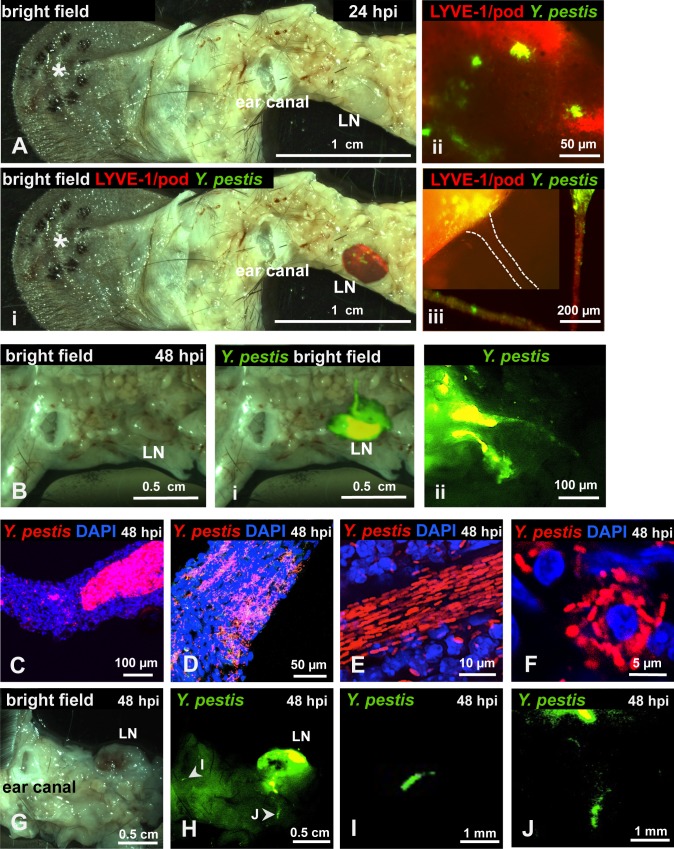
*Y. pestis* in afferent lymphatic vessels and LNs. Mice were inoculated with RFP-*Y. pestis* and the ear pinna was removed along with surrounding tissue, including draining LNs. (A) LN at 24 hpi, asterisk denotes the site of injection; (i) superimposed fluorescence of LN, (ii) microcolonies in LN, (iii) microcolony at the site where afferent lymphatic vessel (dotted lines) connects with LN; in the same panel, two sections of an afferent tube (red) with bacteria (green) are shown. (B) LN at 48 hpi; (i) superimposed fluorescence of LN, (ii) LN with two afferent vessels. (C-E) Bacteria in afferent vessels. (F) Bacteria associated with host cell in afferent vessel. (G-J) Bacteria in afferent lymphatic vessels at sites distant to LN. (G) bright field, (H) superimposed fluorescence showing a highly colonized LN. (I-J) detail of bacteria in vessels at sites distant to LN. C-J images from tissues harvested at 48 hpi. RFP-*Y. pestis* were used to image bacteria. In A, B, and G-J, signal was false-colored green for better visualization; DAPI was used to identify host cell nuclei; Antibodies to LYVE-1 and podoplanin were used to image lymphatic tissue. Experiments were performed a minimum of 3 times and representative pictures are shown.

To determine if bacteria could be detected in the LN and lymphatic vessels that connect ear and LN, we removed the infected ear pinna along with as much adjacent tissue as possible up to the cervical region, including the draining LN and the lymphatic vessels that connect both tissues (S2 Fig.). In LNs at 24 hpi, *Y. pestis* appeared to be in discrete microcolonies and inside afferent lymphatic vessels that attach to the LN (Fig. 2A). At 48 hpi, very strong signal was detected from the same sites (Fig. 2B). Bacteria inside the lymphatic vessels attached to the LN were found either as single cells and small groups or formed tight clumps (Fig. 2C-E). Regardless of how they were arranged (single cells or clumps), the bacteria appeared to follow linear paths (Fig. 2D and E). These paths could be the result of fluid/sheer stress forces and might reflect the inner ‘structure’ of lymphatic vessels. There also were numerous bacterial associations with host cells, as revealed by DAPI staining (Fig. 2F). Lastly, bacteria were detected in the lymphatic vessels that connect the LN and the ear pinna (Fig. 2G-J), at sites distant from the LN (closer to the ear). While detection of bacteria was infrequent at these sites, when observed, bacteria appeared in tight aggregates that delineated the shape of the lymphatic vessel (Fig. 2 I and J). These are direct observations of *Y. pestis* from the tissues through which it disseminates. The use of a low-volume ID model of infection and an imaging approach using whole mounts provided images that depict how fully virulent *Y. pestis* interacts with the host *in vivo*.

### *Y. pestis* passes through a bottleneck during dissemination to the LN that drains the skin

From our previous observations we hypothesized that the majority of bacteria we observed in the ear escape the skin to travel to the LN. To test this, we used a dissemination assay and asked whether all the members of the inoculum population could be found beyond the IS. The dissemination assay was based on the use of 10 oligonucleotide-tagged *Y. pestis* strains. These strains (hereafter referred to as “tagged strains” or simply “strains”) were generated by inserting a unique oligonucleotide tag at a neutral site in the bacterial chromosome [12]. Each tagged strain was found to possess the same level of virulence and growth as un-tagged *Y. pestis*. The frequency of each tagged strain in a tissue was analyzed with the Kluskal-Wallis test. No difference in the mean frequency of a tagged strain was found in LNs (p = 0.9020, 13 independent experiments) or ears (p = 0.9739, 8 independent experiments). Similar results were obtained with the Mann-Whitney statistical test (S1 and S2 Tables). A mix of 9 tagged strains served as the inoculum in our ID model of infection. The tenth strain (A6) served as a negative control. DNA extracted from bacteria recovered at desired time points post-inoculation was subjected to Southern dot blot analysis to determine which strains disseminated from the IS. DNA from the inoculum and from un-tagged *Y. pestis* was used as positive and negative controls, respectively (S3A and B Fig.).

**Figure 3:**
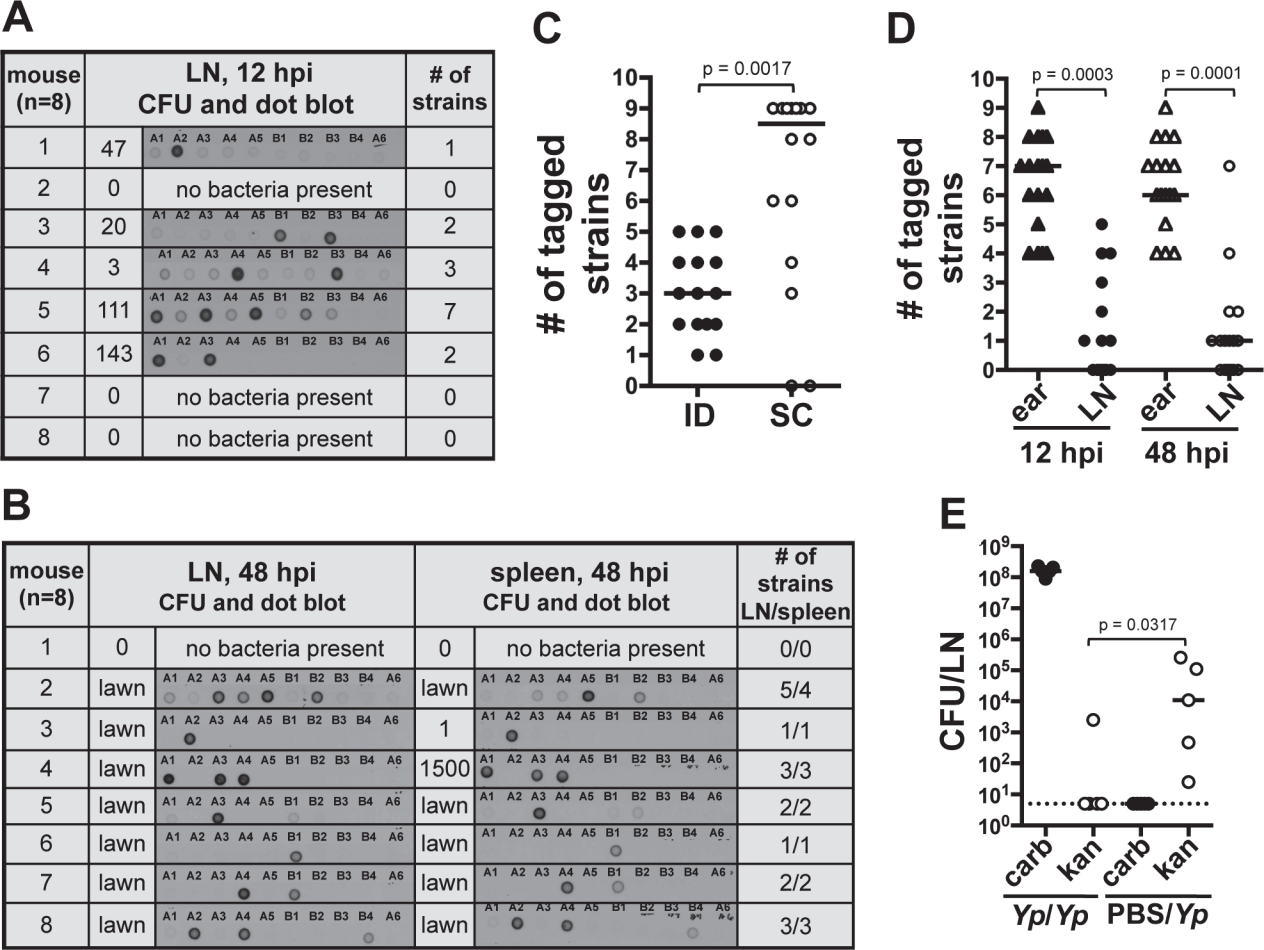
A bottleneck limits dissemination of *Y. pestis* to the draining LN. (A and B) Southern dot blot analysis of DNA from bacteria from (A) LNs harvested at 12 hpi and (B) LNs and spleens at 48 hpi. Each row shows results from a single mouse (identified with a number). The number of CFU obtained from each tissue and from which DNA was extracted is also shown. Representative blots are shown for A and B. A dose of 211 CFU was used. Plates that were completely covered with a layer of bacteria (as opposed to isolated colonies) are identified as “lawn”. (C) Number of tagged strains in LNs after ID (black) or SC (white) inoculation at 48 hpi. A dose of 303 CFU and 331 CFU were used for the ID and SC inoculations, respectively. Each symbol represents a value from an individual mouse. Bars indicate medians per group. Results from two combined experiments are shown. (D) Number of tagged strains in ears (triangles) and LNs [circles at 12 hpi (black) and 48 hpi (white)]. A dose of 226 CFU was used. (E) Bacterial burden from LNs 48 h after the first of two consecutive inoculations. Mice were inoculated first with carbenicillin resistant *Y. pestis* (652 CFU) and, 24 h later, with kanamycin resistant *Y. pestis* (*Yp/Yp*, 449 CFU). Another group of mice was mock inoculated with PBS and, 24 h later, inoculated with kanamycin resistant *Y. pestis* (PBS/*Yp*, 449 CFU). The dotted line represents the limit of detection. Each symbol represents a value from an individual mouse. Bars indicate medians per group. The Wilcoxon matched-pairs signed-rank test (D) or the Mann Whitney test (C) and (E) was used to determine statistical significance and the p value is shown. Differences between groups were considered to be statistically significant when p < 0.05. Experiments were performed a minimum of 3 times and data from representative experiments are shown.

At early time points, 12 hpi, the average number of tagged strains in the LN was 2.2 (range from one to seven tagged strains, Fig. 3A). At 48 hpi, colonization of the LN was well established, systemic dissemination had occurred, and mice were close to succumbing to disease. The average number of tagged strains present in the LN at 48 hpi was 2.8 (range from one to five tagged strains, Fig. 3B). This suggests a bottleneck occurs during the first 12 h of infection, before systemic dissemination takes place. In addition to LNs, we also collected spleens, an organ we use to assess systemic dissemination. All but one mouse (n = 8) had the exact same strain population in spleens and in LNs (Fig. 3B). The mouse that was the exception to this, had strains A3, A4, A5, and B2 in the LN but only A5 and B2 in the spleen. Repetitions of this experiment showed the same trend: either the exact same strains in both organs (majority of cases), or spleens lacking one or more strains that were present in LNs. We never observed a strain present in the spleen that was not present in the LN. This is in agreement with the notion of *Y. pestis* disseminating from IS to LN and then to the rest of the body. More importantly, this suggests the bottleneck defines the population that is responsible for systemic colonization of the host and that will be (potentially) transmitted to a naïve flea.

We then increased the inoculum 10-fold (∼2000 CFU) to test whether increasing the numbers of each tagged strain (∼222 CFU per strain) would alter the number of strains reaching the LN. This inoculum far exceeds the reported average inoculated in mouse skin by a flea (636 CFU, median 82 CFU) [8]. Out of eight mice, four had all 9 tagged strains, one had 8, one had 7, and two had 6 (S3C Fig.). This indicates that even when the number of CFU per tagged strain is considerably higher than what is expected during a natural infection, all the members of an entire strain can still be prevented from reaching the LN. These data indicate colonization of the LN and the rest of the body occurs from only a few bacteria after *Y. pestis* passes through a strong bottleneck.

We also tested whether or not the bottleneck was an ear-specific phenomenon or if it could be replicated from a different anatomical site of the mouse. We performed a dissemination assay comparing mice inoculated in the ear (harvesting the superficial parotid LN) with mice inoculated in the foot (harvesting the popliteal LN). A limited number of tagged strains were observed in the LN (median of 3) and spleens (median of 2) of mice that were inoculated in the foot, indicating that bottleneck formation occurs at anatomical sites other than the ear (S3D Fig.). To determine if the bottleneck was strictly linked to the dermis, we repeated our dissemination assay using a subcutaneous (SC) model of infection [13]. Surprisingly, the median value of tagged strains in LNs from mice inoculated SC was higher (8 tagged strains) than in LNs from mice inoculated ID (3 tagged strains) (Fig. 3C). Furthermore, in half of the mice inoculated SC the bottleneck was completely abrogated. This indicates a key role of the dermis microenvironment in bottleneck formation.

**Bacteria that do not pass through the bottleneck are confined to the skin.** We hypothesized that the source of the bottleneck was bacterial killing in the skin. To test this, we compared the number of strains present in the ear with the number of strains present in the LN of the same mouse. If our hypothesis was correct, the number of strains in the ear should not exceed the number of strains in the LN. Notably, at 12 and 48 hpi, the ear contained a number of strains that ranged between four to nine and that was significantly higher than the number of strains found in the LN (Fig. 3D and S3E Fig.). These results indicate the bottleneck is not due to rapid elimination of bacteria from the skin. These data also indicate bacteria that do not pass through the bottleneck (i.e. do not establish infection in the LN) are confined to the skin throughout infection.

The above data suggest that after deposition in the skin, bacteria elicit a change in the microenvironment of this tissue altering their ability to move to the LN. To test this, we inoculated mice in the ear with a *Y. pestis* strain resistant to carbenicillin and 24 hpi, we inoculated the same animals at the same spot with a *Y. pestis* strain resistant to kanamycin. LNs were harvested 48 h after the first inoculation and plated on media with either carbenicillin or kanamycin. Bacterial loads in LNs were compared to those from a group of mice injected with PBS at the first inoculation time point (control group). We found lower bacterial loads of kanamycin resistant bacteria in LNs of mice whose ears were previously exposed to bacteria in comparison with those that were exposed to PBS (Fig. 3E). This suggests interactions of *Y. pestis* with the skin “activate” this tissue, resulting in detrimental effects to bacteria and a decrease in their ability to escape to deeper tissues. Thus, the bacteria that escape to the LNs, most likely do so before this activated stage takes place.

**Figure 4:**
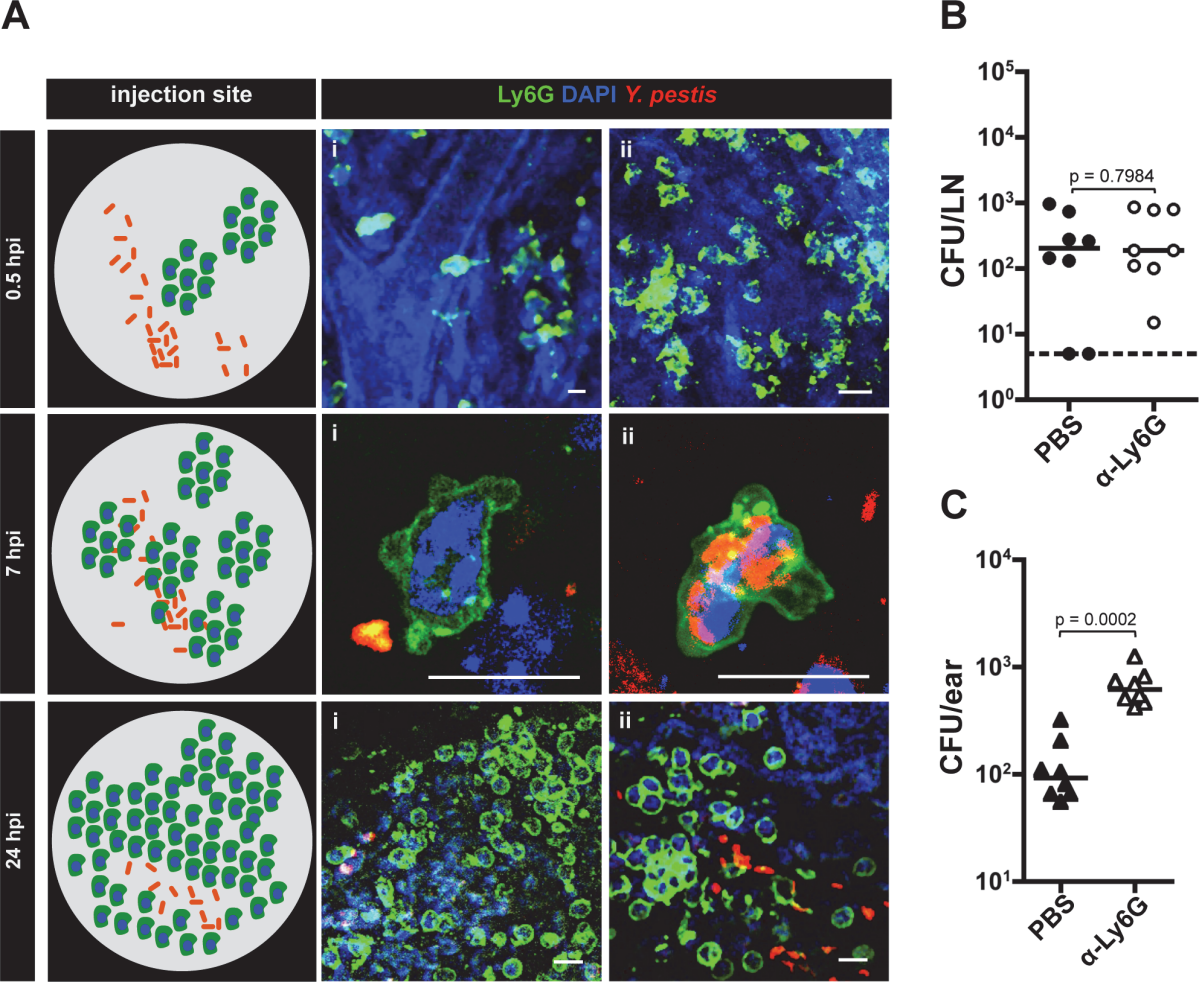
Role of neutrophils during infection. (A) Neutrophils at the IS at different time points. A diagram summarizing the events per time point is shown (left). At 0.5 hpi (i) neutrophil extravasation and (ii) cluster of neutrophils. Bacteria are distant from neutrophils at this time point and are not detected in the same field with neutrophils. At 7 hpi (i) bacteria close to neutrophil and (ii) bacteria inside a neutrophil. At 24 hpi (i) large cluster of neutrophils and (ii) bacteria close to neutrophils. RFP-*Y. pestis* was used to image bacteria; fluorescently labeled antibody to Ly6G was used to image neutrophils (green), and DAPI was used to stain host cell nuclei. At least four ears per time point were analyzed and images shown here belong to representative experiments. The images show maximum intensity projections from z-stacks. Scale bar is 10 μm. (B) Bacterial burden in LN (circles) or (C) ears (triangles) harvested at 12 hpi from mice injected with PBS (black) or with neutrophil depleting antibody to Ly6G (white). For (B) and (C) the doses used were 189 CFU and 206 CFU, respectively. Each symbol represents bacterial burden from a single mouse. Horizontal bars are the median of the group. The dotted line represents the limit of detection. All values obtained from ears were higher than the limit of detection. Statistical significance was determined using the Mann Whitney test and the p value is shown. Differences between groups were considered to be statistically significant when p < 0.05. Experiments were performed a minimum of 2 times and data from one representative experiment are shown.

**Neutrophils control *Y. pestis* proliferation in the dermis**. We thought that “activation” of the skin could derive from recruitment of cells of the innate immune response. Recent work suggested neutrophils are important during *Y. pestis* infection in the skin [10,14] but whether these cells play a role during subsequent infection steps is unknown. To gain insights into the role of neutrophils during infection, we tracked these cells by microscopy imaging using fluorescently labeled α-Ly6G, an antibody that binds to neutrophils [15]. At 30 minutes post inoculation, very few isolated clusters of neutrophils were observed in the skin (Fig. 4Ai and Aii). These clusters were distant from the bacteria, as bacteria and neutrophils were seen a few ‘fields of view’ apart from each other. This was distinctly different from later time points when bacteria and neutrophils could be visualized in the same field of view. Between 4 and 8 hpi, many bacteria were in close proximity to neutrophils or inside them (as determined by confocal microscopy, S4 Fig.). At 24 hpi a prominent increase in neutrophils was observed in comparison to previous time points. Neutrophils were highly concentrated at the injection site, forming very dense clusters. However, very few bacteria seemed to be associated with neutrophils at this later time point.

We and others have speculated that neutrophils could contribute to bacterial trafficking to LNs [10]. However, depletion using a neutrophil specific depletion antibody (α-Ly6G, S5A Fig.) had no impact on bacterial trafficking (Fig. 4B), indicating neutrophils do not contribute significantly to this process. Neutrophil contributions to bottleneck formation were also assessed. Compared to mice injected with PBS, injection of α-Ly6G resulted in an increase in the number of tagged strains in the LN. This increase, while reproducible, was not statistically significant (S5B Fig.), which supports results from CFU data that indicate neutrophils are not needed for trafficking. However, we realized from our microscopy observations that substantial recruitment of neutrophils to the inoculation site occurred, and that bacterial numbers at this site did not seem to change over time. Thus, we hypothesized that neutrophils control bacterial burden in the skin without being able to clear infection. To test this, we compared bacterial burden in the skin (ear pinna) in mice treated with neutrophil depleting antibody or mock treated with PBS. Mice treated with neutrophil depleting antibody showed a highly significant increase in the number of bacteria in the skin when compared to mock treated mice (Fig. 4C). Overall, these experiments suggest *Y. pestis* interacts with neutrophils in the dermis and that these interactions severely restrict bacterial proliferation in the skin.

**Figure 5:**
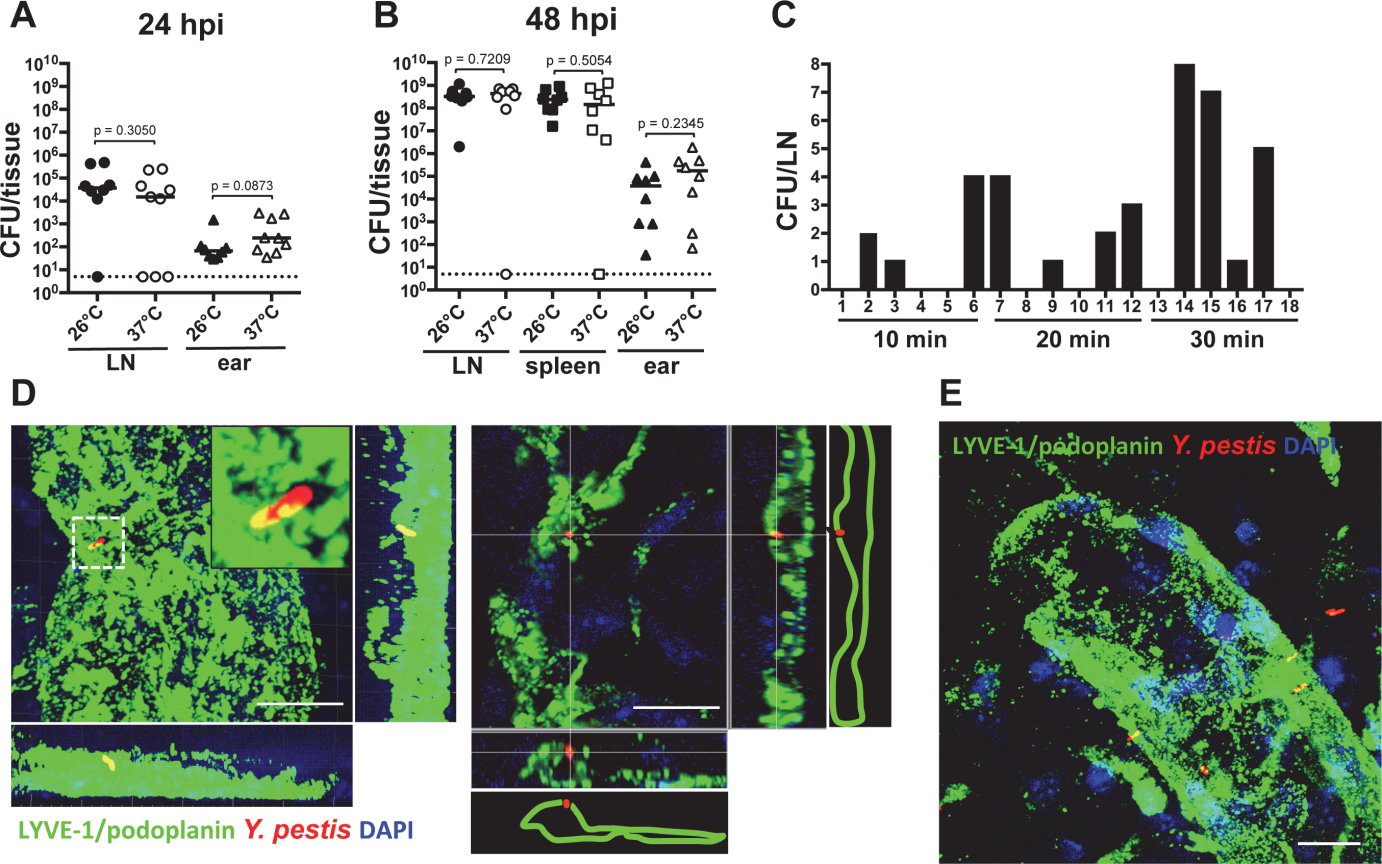
Bacterial dissemination from the ear to the draining LN. (A) Bacterial burden in LN (circles) and ears (triangles) harvested at 24 hpi from mice inoculated with bacteria grown at 26°C (225 CFU, black) or 37°C (283 CFU, white). (B) Bacterial burden in LN (circles), spleens (squares) and ears (triangles) harvested at 48 hpi from mice inoculated with bacteria grown at 26°C (453 CFU, black) or 37°C (290 CFU, white). For (A) and (B), each symbol represents bacterial burden from a single mouse. Horizontal bars are the median of the group. The dotted line represents the limit of detection. Statistical significance was determined using the Mann Whitney test and the p value is shown. Differences between groups were considered to be statistically significant when p < 0.05. (C) Bacterial burden in LNs at different time points after inoculation with 337 CFU. Each bar represents the value from an individual LN (18 LNs were analyzed). A representative experiment is shown. (D) Bacterium associated with lymphatic vessel (green) at 15 min post inoculation. Maximum intensity projection (left) and xz slice (right). (E) Maximum intensity projection of bacteria associated with lymphatic vessel (green) at 20 min after inoculation. Scale bar is 10 μm. RFP-*Y. pestis* were used to image bacteria; DAPI was used to identify host cell nuclei; fluorescently labeled antibody against LYVE-1 was used to image lymphatic vessels. See S1 Video. Experiments were performed a minimum of 2 times and data from representative experiments are shown.

**Dissemination of *Y. pestis* in a phagocyte-independent manner**. It is currently thought that for *Y. pestis* to move into LNs, an intracellular stage must exist [6,7]. However, results presented here and another recent study [10] suggest neutrophils are not important for bacterial movement to LN. In addition, Shannon, et al. concluded that the interactions of *Y. pestis* with dendritic cells *in vivo* are minimal and unlikely to be significant for bacterial dissemination [10]. We explored the notion that bacteria could be transported in a phagocyte-independent manner. *In vivo* models of bubonic plague infections use *Y. pestis* grown at 26°C, a temperature consistent with delivery from a flea. At 26°C, the antiphagocytic factors that are crucial for *Y. pestis* survival in the host are predicted to be weakly expressed. This is supported by studies with cell lines and primary cells that report *Y. pestis* is significantly less susceptible to phagocytosis by macrophages, dendritic cells, and to a lesser extent by neutrophils, when grown at 37°C than when grown at 26°C [16–18]. Because it has been predicted that *Y. pestis* travels from the skin to LNs inside of phagocytes, we expected that bacteria grown at 37°C would not disseminate efficiently to LNs. Surprisingly, we found that mice inoculated with comparable numbers of bacteria grown either at 26°C or 37°C showed no difference in bacterial loads in LNs at 12 hpi (S6 Fig.) or in LN and ears at 24 hpi (Fig. 5A). Comparable results were obtained in LNs, spleens, and ears at 48 hpi (Fig. 5B). These data indicate bacteria that are not susceptible to phagocytosis are as efficient in reaching the LN as bacteria that are susceptible to phagocytosis.

Before this study, we always harvested *Y. pestis* from LNs several hours after inoculation. This is mainly because we assumed bacteria would move inside a phagocyte and movement of these host cells is slow, taking a few hours. Given the results presented here we wanted to test whether *Y. pestis* could reach LNs within minutes after injection, as happens with small molecules such as Evan’s blue (S2 Fig.). After injection into the skin, Evan’s blue can be found in LNs within 30 min as it moves with the flow of lymph, without the need of phagocytic cells. Thus, LNs were harvested at 10, 20 and 30 min after bacterial inoculation in the ear. We detected *Y. pestis* in the LN as early as 10 min after inoculation (Fig. 5C), indicating the bacteria can reach the LN as fast as small molecules that travel through lymphatic vessels with the flow of lymph.

Lastly, we wanted to determine whether bacteria that were associated with lymphatic vessels of the ear were also associated with phagocytic cells. To this end, we imaged RFP-*Y. pestis* during the first 30 min post inoculation, after immunofluorescence labeling of lymphatic vessels of the ear. As determined by DAPI staining, bacteria in close proximity or associated with lymphatic vessels were not associated with any host cells (Fig. 5D and E, S7 Fig. and S1 Video). Together, these experiments indicate that in our infection model, association with phagocytes is not necessary for *Y. pestis* to disseminate from the skin to the draining LN.

## Discussion

Pathogen dissemination in the host is a crucial and understudied process of infection. Although many studies have addressed bacterial-host interactions of *Y. pestis* (and other highly virulent pathogens), most of them are limited by the use of *in vitro* approaches and/or of attenuated strains. While many noteworthy observations have derived from these studies, we think that a complete picture of infection can only be achieved by also examining fully virulent strains in an *in vivo* context. Herein, we looked at dissemination of fully virulent *Y. pestis* in mice using three approaches: (a) an ID model of infection with minimal disruption to the skin and a low dose of bacteria to mimic delivery to the dermis that occurs during fleabite, (b) an assay that allowed us to follow dissemination within the host at a population level, and (c) microscopic imaging of the skin and underlying tissue to observe host-pathogen interactions at a cellular level. The combination of these strategies allowed us to make biologically relevant observations of the steps that follow bacterial entrance into the body and that define dissemination. The use of an ID model of infection is of particular relevance as hematophagous insects probe the dermal layer of the skin [19]. Moreover, histological sections of skin probed by infected fleas show *Y. pestis* is deposited into the dermis and not into the subcutaneous space [11]. Notably, our experiments show *Y. pestis* escape from the skin is restricted and that neutrophils play a role in controlling bacterial proliferation in this tissue. More importantly, we show bacteria that are not cell-associated enter lymphatic vessels and appear in LNs within minutes after inoculation. Our observations are not consistent with bacterial trafficking within phagocytes. While intracellular trafficking might occur at later stages, our data indicate the first bacteria that arrive in the LN do not need phagocytic cells to reach this compartment.

Upon ID injection, the dissemination assay revealed a bottleneck that accounts for a previously unrecognized barrier for *Y. pestis* to disseminate from the IS. The bottleneck has strong evolutionary implications for *Y. pestis*, because it defines the population of bacteria that have the potential to be acquired by a naïve flea and thus be transmitted to a new host. More importantly, the bottleneck reveals even highly virulent pathogens, such as *Y. pestis*, encounter barriers that affect dissemination efficiency. Nearly 10% of our mice showed no bacteria in the LN. This was not due to ineffective inoculation as we confirmed that 100% of the mice had bacteria in the inoculated ear. This suggests efficient bottlenecks form in a fraction of immunocompetent individuals. Efficient bottlenecks in a few individuals could occur in part as a result of inherent variability in the immune responses of a population. It is not known how a natural flea inoculation, in comparison with needle inoculation, would affect bottleneck formation. In *Leishmania major* infections, a more robust and prevalent immune response is observed during sand fly versus needle inoculations [20]. However, in contrast to what was observed for *Leishmania*, no difference was observed in host responses to flea compared to needle inoculation of *Y. pestis* [21]. Therefore, if fleas deposit a relatively low dose of *Y. pestis*, one would expect the rate of successful infections from fleabites would be low, due to the bottleneck. In agreement with this, studies using fleas estimate the rate of successful plague infections in mice by flea bites to be less than 50% [8].

Interestingly, the bottleneck is abrogated when bacteria are delivered in the SC space beyond the dermis. This observation is relevant as it is very likely fleas deliver *Y. pestis* into the dermis and not the SC space [11,19,22]. The dermis is particularly proficient in triggering immune responses against invaders. Differences in immunogenicity between layers of the skin also have been shown to exist in cancer research; adenocarcinoma cells in rats develop into tumors only after SC but not ID injections [23]. The SC layer of the skin, on the other hand, is less immunologically competent than the dermis and this might facilitate the passage of more tagged strains into the LN [24]. This might occur due to a delayed influx of immune cells in SC tissue from blood therefore allowing for more local bacterial replication prior to dissemination.

Very few studies have used imaging to probe host-pathogen interactions during cutaneous infections *in vivo* [19]. The use of an ID infection model and fluorescence confocal microscopy of unsectioned tissues provided us with high-resolution observations to reveal bacterial localization and associations in the host without requiring the use of attenuated strains. Microscopy imaging provides qualitative information to understand interactions with the host that is impossible to collect by traditional approaches using bacterial counts from harvested organs. Recent research suggested bacteria could evade a strong neutrophil response in the skin [10,25]. Our observations are in agreement with these reports, as we did not observe bacterial clearance in the skin. However, we also found that neutrophils severely restrict bacterial colonization of the skin, and thus, revealed a role of neutrophils *in vivo*. The bacterial restrictive properties of the skin are likely to be absent at the onset of infection. However, once present, they affect the bacteria in the skin in such a way that movement of newly inoculated bacteria into LNs is restricted, as shown by our double inoculation experiment.

How pathogens disseminate from the IS into deeper tissues is one of the most relevant questions in microbial pathogenesis and one that is very difficult to address using direct approaches. For many pathogens, including *Y. pestis*, an intracellular stage to reach distant tissues has been proposed [6,7]. In this study, our data suggest association with host cells might not be necessary for *Y. pestis* to travel to LNs. Very few studies have addressed this question, but similar claims have been made for the highly virulent pathogen *Bacillus anthracis* [26] and for *Salmonella abortusovis* [27]. In the latter case, 80% of the bacteria traveling to the LN were found to be free in lymphatic vessels during the first 90 minutes of infection. While bacteria might travel to LNs in multiple ways, our data suggest that an intracellular stage is not required.

Finally, we think that a phagocyte-independent mechanism for bacterial movement might explain bottleneck formation. This is because in a phagocyte-independent mechanism, only the very few bacteria that remain in suspension and do not adhere to the skin can be moved to the LN with the flow of lymph. In addition to the inability of all bacteria to be ‘taken’ by the flow of lymph and be conducted to a lymphatic vessel, bottleneck formation could also result from one or the combination of the following: (a) neutrophils might contribute to some extent to the bottleneck if bacteria encounter any of these cells during movement through the lymphatic vessels, and (b) phagocytic cells in the LN may contribute by killing newly arrived bacteria. Further understanding of the cause(s) of the bottleneck is the subject of future studies.

## Materials and Methods

### Ethics statement

This study was carried out according to the recommendations in the Guide for Care and Use of Laboratory Animals of the National Institutes of Health. All animal studies were approved by the Institutional Animal Care and Use Committee of the University of North Carolina at Chapel Hill, protocol 11–128. All efforts were made to minimize suffering; animals were monitored every 12 h following infections and were euthanized upon exhibiting signs of morbidity.

### Bacterial strains and culture conditions

Fully virulent *Y. pestis* CO92 [28] was used in all experiments. For the dissemination assay *Y. pestis* was tagged with 10 variants of an oligonucleotide signature tag [12]. The oligonucleotide tag, along with a kanamycin resistance cassette, was inserted at the Tn7 *att* site in the bacterial chromosome [29]. Each tag contains a unique sequence of ∼80 bp flanked by invariant sequences that were used for amplification. The 10 tagged *Y. pestis* CO92 strains were tested in our animal models to ensure each had retained the same virulence characteristics of the parent strain. For the dissemination assay, tagged strains were cultured in brain and heart infusion broth (BHI, BD Biosciences, Bedford MA) with kanamycin and incubated at 26°C unless otherwise stated. Standardized liquid cultures (based on optical density at 600 nm) were mixed in a single tube and the mix was serially diluted in phosphate buffered solution (PBS) to obtain the desired inoculum. Methods for detection of the tagged strains are described below. For the double inoculation experiments, the carbenicillin resistant strain also expressed *rfp* (for easy detection). A mix of the tagged strains was used as the kanamycin resistant strain. For the experiments with bacteria grown at 37°C, liquid cultures were grown in BHI with 2.5 mM CaCl_2_ for 6 h at 26°C and then shifted to 37°C for 12.5 h [30]. Where needed, kanamycin (kan) was added at 25 μg/ml and carbenicillin (carb) at 100 μg/ml.

### Animal infections

Six-to-eight week-old female C57BL/6J mice (Jackson Laboratory, Bar Harbor, ME) were inoculated under anesthesia (ketamine/xylazine). ID inoculations were done in the dorsal side of the ear pinna or the upper side of the foot. A volume of 2 μL was inoculated with the aid of a Pump11 Elite syringe pump (Harvard Apparatus, Holliston, MA) and a SURFLO winged infusion set with a 27-gauge needle (Terumo, Lakewood, CO). SC inoculations were performed as previously described [13] injecting a volume of 2 μL. Animals were sacrificed by injection with sodium pentobarbital. Organs were harvested at different time points and homogenized in PBS. Homogenates were serially diluted and plated on BHI agar and incubated at 26°C for 48 h to obtain bacterial counts. Mann Whitney or Wilcoxon matched pairs signed rank tests were used for statistical analysis, establishing statistical significance at p < 0.05 using GraphPad Prism version 4.0c (GraphPad Software, La Jolla, CA).

### Whole ear imaging

Mice were inoculated with *gfp*- or *rfp*-expressing *Y. pestis* [31,32] following the procedures described above. After the mice were sacrificed, their ears were separated from the head, gently punctured with scissors (to facilitate fixative diffusion), and submerged in 10% buffered formalin for 24 h. The dermis of the dorsal leaflet was exposed by separating both leaflets and removing the layer of cartilage that separates them. Ears were mounted on glass slides with ProLong Gold antifade reagent with 4′, 6-diamidino-2-phenylindole (DAPI; Molecular Probes, Eugene, OR). Neutrophils were stained by tail-vein injection of fluorescently labeled antibodies against Ly6G (BD, Franklin Lakes, NJ). Lymphatic vessels were stained by ID injection of fluorescently labeled podoplanin antibody clone 8.1.1 (BioLegend, San Diego, CA) and LYVE1 antibody (Fitzgerald, Acton, MA) [31,33]. For all antibodies, 5 ng of antibody was used per mouse. Images were taken with an Olympus FV1000 MPE SIM laser scanning confocal microscope and analyzed with the Fiji (ImageJ 1.48t) software package [15,33,34]. The same procedures were used to image LNs and the lymphatic vessels that connect ears and LNs. These vessels were also visualized by injection of 10% Evan’s blue in the ear.

### Detection of oligonucleotide-tagged strains

Bacteria that grew on agar plates from undiluted homogenized organs were mixed with 1 mL of PBS using a bacterial cell spreader until a homogeneous suspension was formed. A volume of 20 μL of this suspension was added to BHI broth with kanamycin and incubated at 26°C in a roller drum for 14 h. When less than 15 colonies were present on a plate, individual colonies were picked with a wooden stick and grown under the same conditions described. DNA was extracted from liquid cultures using a Wizard Genomic Purification Kit (Promega, Madison, WI). DNA concentrations were measured and standardized at 100 ng/μL. The oligonucleotide tag sequence was amplified by PCR using primers P2 (5’-TAC CTA CAA CCT CAA GCT-3’) and P4 (5’-TAC CCA TTC TAA CCA AGC-3’), which hybridize to the invariable region of all oligonucleotide tags. PCR reactions were prepared under standard conditions except for (a) the use of a mix of dideoxynucleotides (ddNTPs) with a ddATP:ddCTP:ddGTP:ddTTP ratio of 5:1:5:5 and (b) ddCTP labeled with ^32^P was incorporated into the reaction. PCRs were cleaned using a MiniElute kit (Promega, Madison, WI). In this manner, this ^32^P-labeled amplicon consisted of a mix of DNA from any of the tagged strains that was present in a sample. Southern dot blot assays were conducted using the obtained labeled amplicon as probe. The probe was hybridized to positively charged nylon membranes (Roche, Manheim, Germany) previously crosslinked with 200 ng of plasmid DNA (pCIITN7K-a) containing the tags, as previously described [12]. The DNA crosslinked to the nylon membrane was arranged in 10 separate spots, each one containing DNA of each of the unique oligonucleotide tags used in the study. Because 10 of the oligonucleotides were used in the membranes and 9 were used to inoculate the mice, one of the oligonucleotides (A6) spotted on the membranes served as a negative control. Photographic film was exposed to the membranes with hybridized DNA and the developed film was scanned to obtain a digital image. The digital image was processed using the Fiji (ImageJ 1.48t) software package [33] to invert the image (‘invert’ function) and to calculate the integrated density value per dot. An integrated density value six times higher than that of the negative control (A6), after background subtraction (‘subtract background’ function), was scored as positive.

### Phagocyte depletion

Ly6G+ cells were depleted after tail vein injection of 100 μL (0.2 mg/mL) of low endotoxin Ly6G antibody clone 1A8 (BioLegend, San Diego, CA) [15,34]. Antibody to Ly6G was injected 24 h before inoculation of bacteria. Depletion of targeted cells was assessed by flow cytometry defining neutrophils as Ly6G+ cells; monocytes as F4/80−, CD11b+, Ly6G− cells; macrophages as F4/80+ cells; and dendritic cells as F4/80−, CD11b+ cells.

## Supporting Information

S1 Fig Intradermal inoculation in the ear pinna. (A) Ear pinna. The black dots denote the edges of the injection site (defined by a transient wheal that forms in the skin immediately after injection and marked by a dotted line). (B) Bright field and (C) fluorescence micrographs of the injection site 8 hours after inoculation with GFP-*Y. pestis*. A high inoculum (∼1500 CFU) was used to enhance signal at low magnifications. White arrowheads mark the position of bacteria to avoid confusion with background fluorescence. Bar for A is 0.5 cm. Bars for B and C are 0.5 mm.
(TIF)

S2 Fig Afferent lymphatic vessels that drain the ear pinna into the superficial parotid LN. (A-D) Lymphatic vessels (blue, from injection of Evan’s blue) run from the ear to the superficial parotid LN. Magnifications (identified with arrowheads in A) are shown in B, C, and D. (D) Afferent lymphatic vessel connected to the LN. The image was taken 20 min after injection of Evan’s blue. The scale bars are 50 μm.
(TIF)

S3 Fig Dissemination assay controls, strength of bottleneck, and bottleneck from the foot. (A) *Y. pestis* was tagged with an oligonucleotide signature tag inserted in the bacterial chromosome at a neutral site. Ten unique tags were used to make ten tagged *Y. pestis* strains (each identical to one another and to un-tagged *Y. pestis* except for the inserted tag). A mix of the tagged strains was inoculated ID in the ear pinna (A6 was left out to serve as a negative control). DNA from bacteria obtained from harvested tissues was used for Southern dot blot. (B) Southern dot blot showing negative and positive controls. DNA from untagged *Y. pestis* was used as a negative control. DNA from the inoculum (2 μL containing nine tagged strains and plated directly from the needle that was used to inoculate mice) was used as positive control. (C) Dot blot from LN of mice inoculated with ∼2000 CFU. Each row belongs to a single mouse (identified with a number). The number of missing tagged strains in each LN is shown. (D) Number of tagged strains in superficial parotid LN (black circles) after ID inoculation (∼200 CFU) in the ear and in popliteal LNs (white circles) and spleens (white squares) after ID inoculation (∼200 CFU) in the upper part of the foot. Organs were harvested at 48 hpi. Each symbol represents the number of tagged strains obtained from a tissue from a single mouse. Horizontal bars represent the median value of the group. (E) Dot blot analysis of ears, LNs, and spleens, at 48 hpi following ID inoculation with ∼200 CFU. Each row shows results from a single mouse. Experiments were performed a minimum of 2 times and data from representative experiments are shown.
(TIF)

S4 Fig *Yersinia pestis* interactions with neutrophils in the skin. (A) *Y. pestis* in close proximity to a neutrophil (green with blue nucleus). (B) A single *Y. pestis* bacterium inside a neutrophil. For (A) and (B), maximum intensity projections are shown. (C) xy slices from the image depicted in (B). The slices advancing into the bottom of the cell (from left to right) through the z-axis of a z stack and show the bacterium is inside the neutrophil and not on its surface. Scale bar is 10 μm. Images taken from ears harvested at 4 hpi. RFP-*Y. pestis* were used to image bacteria; DAPI was used to identify host cell nuclei; and an antibody against Ly6G was used to image neutrophils. Representative images from 3 independent experiments are shown.
(TIF)

S5 Fig Depletion of neutrophils with an antibody against Ly6G. (A) Mean percentage of Ly6G^+^ cells (neutrophils) in mice injected intravenously (via tail vein) with PBS (gray) or an antibody against Ly6G (white). Data from two combined experiments (3 animals per experiment) are shown. Error bars are standard error of mean (SEM). (B) Number of tagged strains in LNs harvested at 12 hpi after ID inoculation in mice injected with PBS (black) or α-Ly6G (white). The dose used was 189 CFU. Each symbol represents a value from an individual mouse. The horizontal bars indicate medians per group. The Mann Whitney test was used to determine statistical significance. Differences between groups were considered to be statistically significant when p < 0.05. Data from a representative experiment is shown.
(TIF)

S6 Fig Bacterial dissemination from the ear to the draining LN. Bacterial burden in LN harvested at 12 hpi from mice inoculated with bacteria grown at 26°C (black) or 37°C (white). A dose of 294 CFU was used for the bacteria grown at 26°C and 269 CFU for the bacteria grown at 37°C. Each symbol represents bacterial burden from a single mouse. Horizontal bars are the median of the group. The dotted line represents the limit of detection. Statistical significance was determined using the Mann Whitney test and the p value is shown. Differences between groups were considered to be statistically significant when p < 0.05. Exposure to bacteria grown at 26°C and 37°C were conducted separately and a single time.
(TIF)

S7 Fig *Yersinia pestis* and lymphatic vessels during the first minutes of infection. Bacterium (red) in close proximity to a lymphatic vessel (green) 10 min post inoculation. No blue signal (DAPI) is detected near the bacterium. Maximum intensity projection (left) and xz slice (right). Scale bar is 10 μm. Experiments were performed a minimum of 2 times and data from representative experiments are shown. RFP-*Y. pestis* were used to image bacteria; DAPI was used to identify host cell nuclei; α-LYVE-1 was used to image lymphatic vessels.
(TIF)

S1 Table Frequency of each tagged strain in LNs. The median frequency of a tagged strain in LNs was compared to the equivalent median of each of the other tagged strains. Data from 13 independent experiments were used in the analysis. The table shows the p values obtained after using the Mann-Whitney statistical test to determine whether or not differences between medians were significant. Differences between medians were determined as significant when p < 0.05. Note that only differences between A6 (negative control) and any of the other tagged strains are significant.
(PDF)

S2 Table Frequency of each tagged strain in ears. The median frequency of a tagged strain in ears was compared to the equivalent median of each of the other tagged strains. Data from 8 independent experiments were used in the analysis. The table shows the p values obtained after using the Mann-Whitney statistical test to determine whether or not differences between medians were significant. Differences between medians were determined as significant when p < 0.05. Note that only differences between A6 (negative control) and any of the other tagged strains are significant.
(PDF)

S1 Video *Yersinia pestis* in close proximity to a lymphatic vessel. This video corresponds to Fig. 5E and depicts bacteria associated with a lymphatic vessel (green). RFP-*Y. pestis* were used to image bacteria; DAPI was used to identify host cell nuclei; fluorescently labeled antibody against LYVE-1 was used to image lymphatic vessels.
(MOV)
