# Supplementary figures and images for: Dissemination of a Highly Virulent Pathogen: Tracking The Early Events That Define Infection
Source: PLoS Pathog. 2015 Jan 22;11(1):e1004587. doi: 10.1371/journal.ppat.1004587 (PMC4303270; doi:10.1371/journal.ppat.1004587)

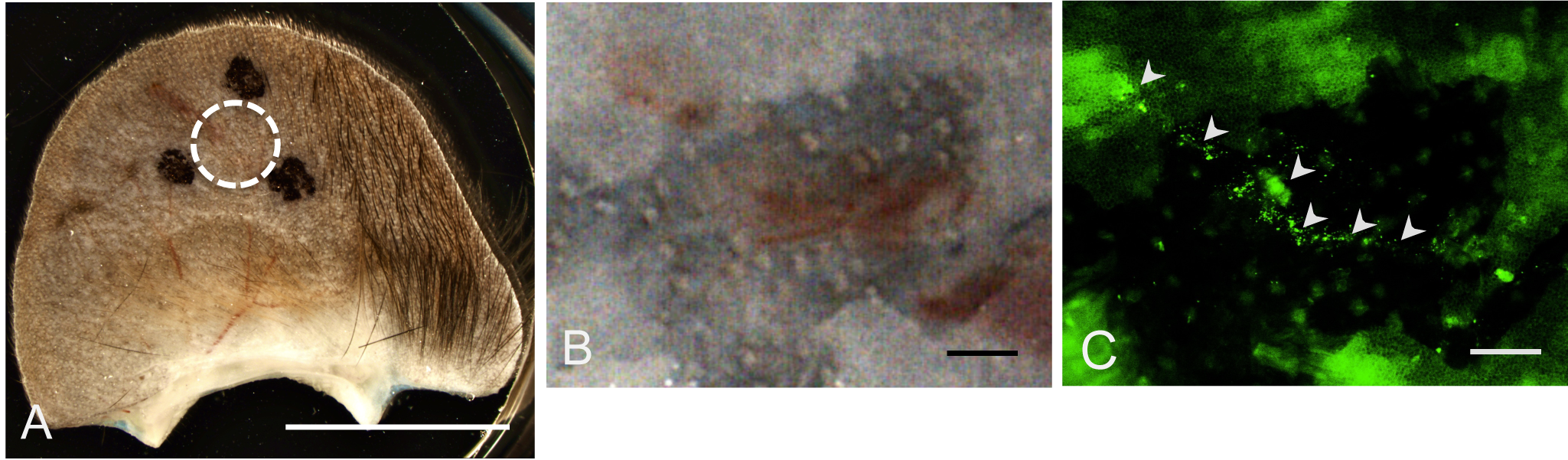

Supplement: S1 Fig — (A) Ear pinna. The black dots denote the edges of the injection site (defined by a transient wheal that forms in the skin immediately after injection and marked by a dotted line). (B) Bright field and (C) fluorescence micrographs of the injection site 8 hours after inoculation with GFP-Y. pestis. A high inoculum (∼1500 CFU) was used to enhance signal at low magnifications. White arrowheads mark the position of bacteria to avoid confusion with background fluorescence. Bar for A is 0.5 cm. Bars for B and C are 0.5 mm. (TIF) [file ppat.1004587.s001.tif]

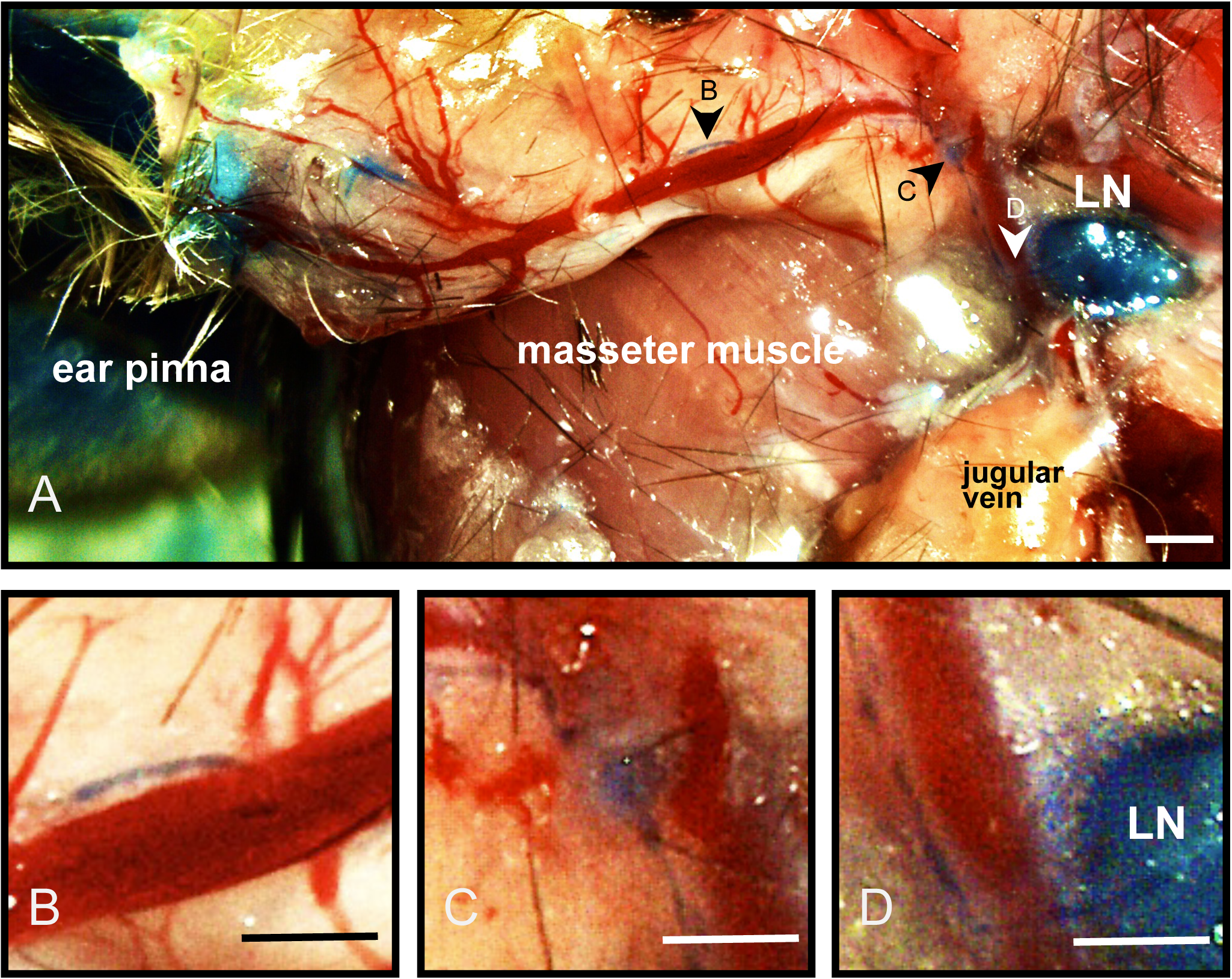

Supplement: S2 Fig — (A-D) Lymphatic vessels (blue, from injection of Evan’s blue) run from the ear to the superficial parotid LN. Magnifications (identified with arrowheads in A) are shown in B, C, and D. (D) Afferent lymphatic vessel connected to the LN. The image was taken 20 min after injection of Evan’s blue. The scale bars are 50 μm. (TIF) [file ppat.1004587.s002.tif]

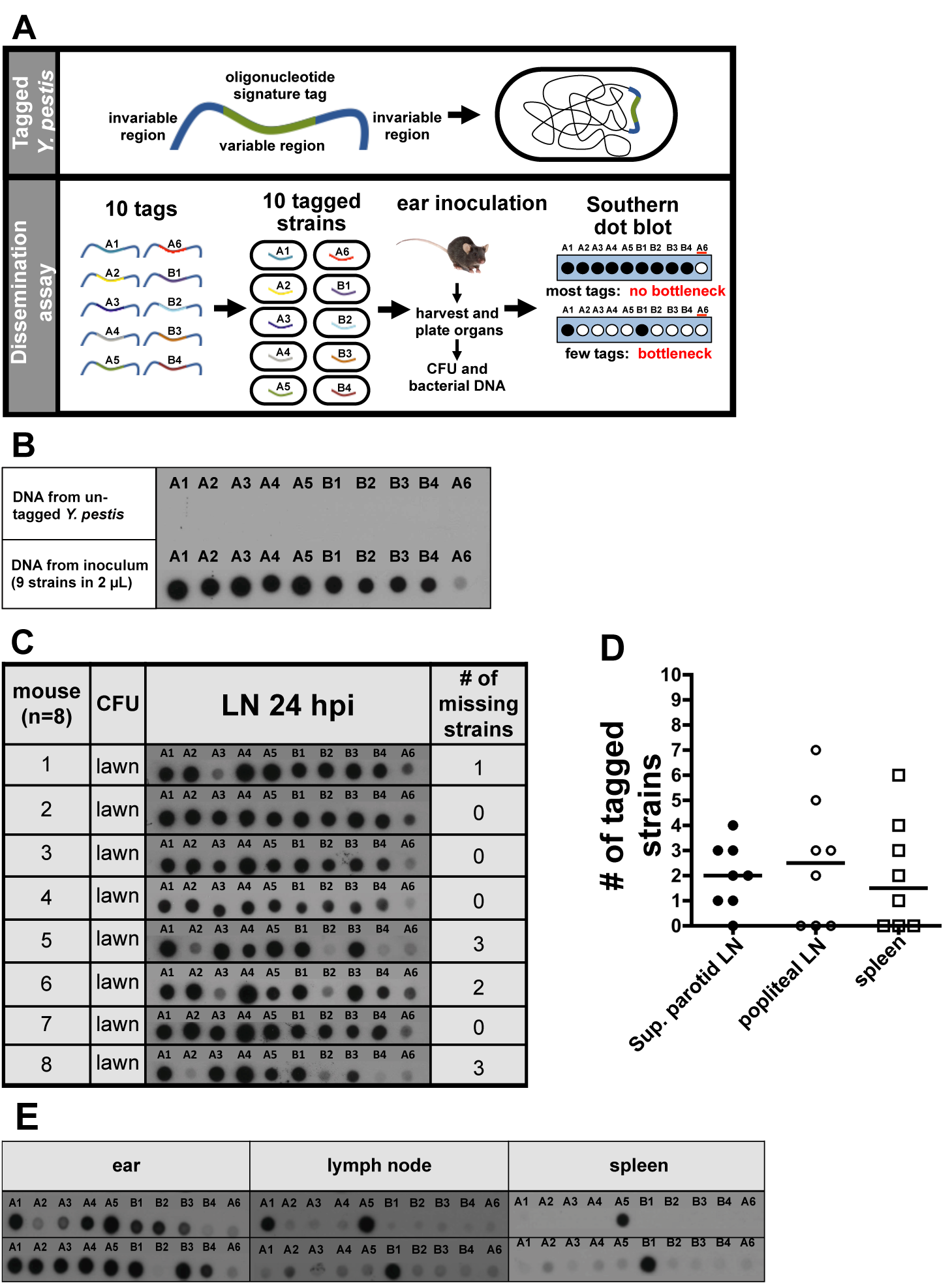

Supplement: S3 Fig — (A) Y. pestis was tagged with an oligonucleotide signature tag inserted in the bacterial chromosome at a neutral site. Ten unique tags were used to make ten tagged Y. pestis strains (each identical to one another and to un-tagged Y. pestis except for the inserted tag). A mix of the tagged strains was inoculated ID in the ear pinna (A6 was left out to serve as a negative control). DNA from bacteria obtained from harvested tissues was used for Southern dot blot. (B) Southern dot blot showing negative and positive controls. DNA from untagged Y. pestis was used as a negative control. DNA from the inoculum (2 μL containing nine tagged strains and plated directly from the needle that was used to inoculate mice) was used as positive control. (C) Dot blot from LN of mice inoculated with ∼2000 CFU. Each row belongs to a single mouse (identified with a number). The number of missing tagged strains in each LN is shown. (D) Number of tagged strains in superficial parotid LN (black circles) after ID inoculation (∼200 CFU) in the ear and in popliteal LNs (white circles) and spleens (white squares) after ID inoculation (∼200 CFU) in the upper part of the foot. Organs were harvested at 48 hpi. Each symbol represents the number of tagged strains obtained from a tissue from a single mouse. Horizontal bars represent the median value of the group. (E) Dot blot analysis of ears, LNs, and spleens, at 48 hpi following ID inoculation with ∼200 CFU. Each row shows results from a single mouse. Experiments were performed a minimum of 2 times and data from representative experiments are shown. (TIF) [file ppat.1004587.s003.tif]

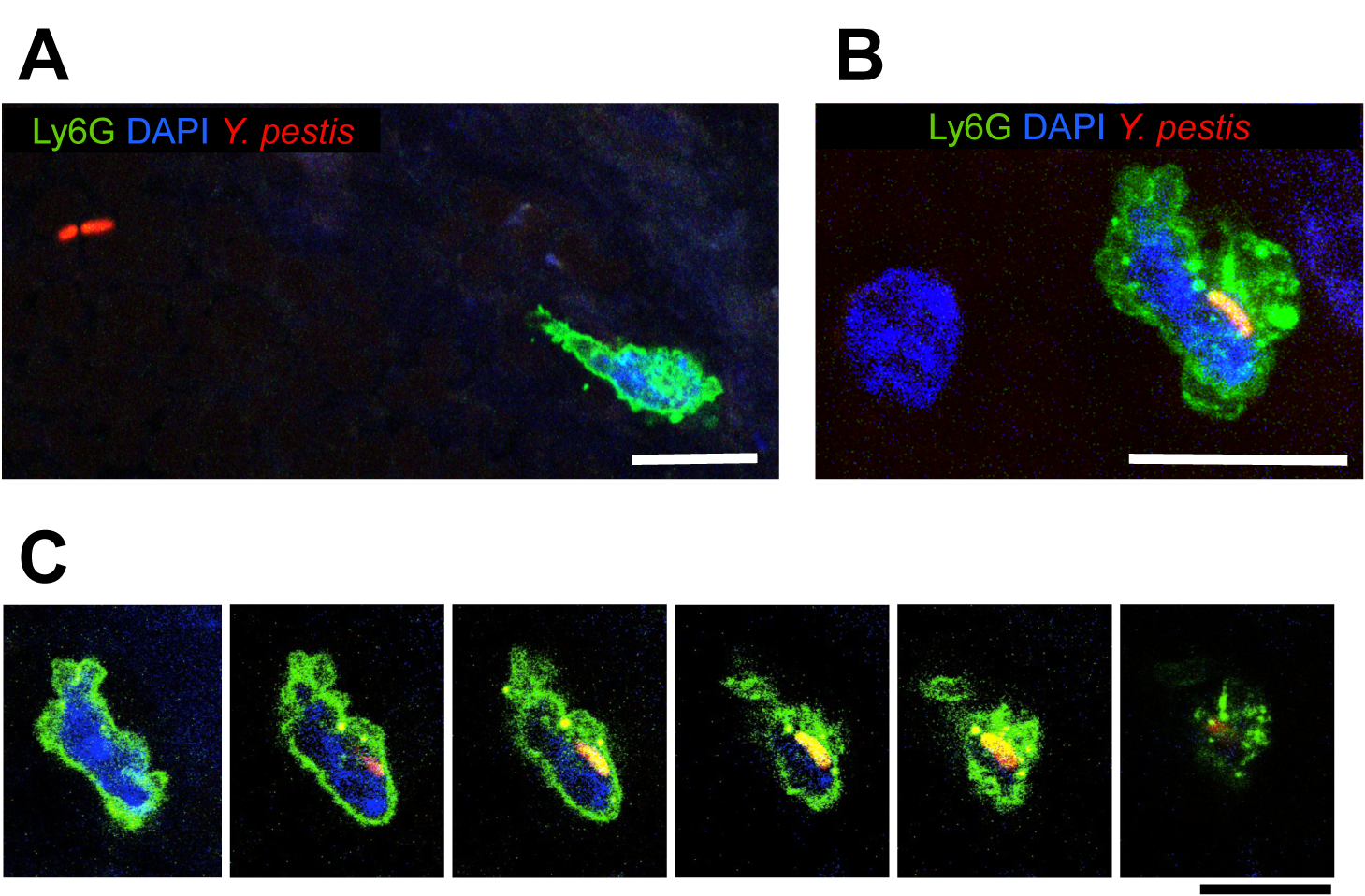

Supplement: S4 Fig — (A) Y. pestis in close proximity to a neutrophil (green with blue nucleus). (B) A single Y. pestis bacterium inside a neutrophil. For (A) and (B), maximum intensity projections are shown. (C) xy slices from the image depicted in (B). The slices advancing into the bottom of the cell (from left to right) through the z-axis of a z stack and show the bacterium is inside the neutrophil and not on its surface. Scale bar is 10 μm. Images taken from ears harvested at 4 hpi. RFP-Y. pestis were used to image bacteria; DAPI was used to identify host cell nuclei; and an antibody against Ly6G was used to image neutrophils. Representative images from 3 independent experiments are shown. (TIF) [file ppat.1004587.s004.tif]

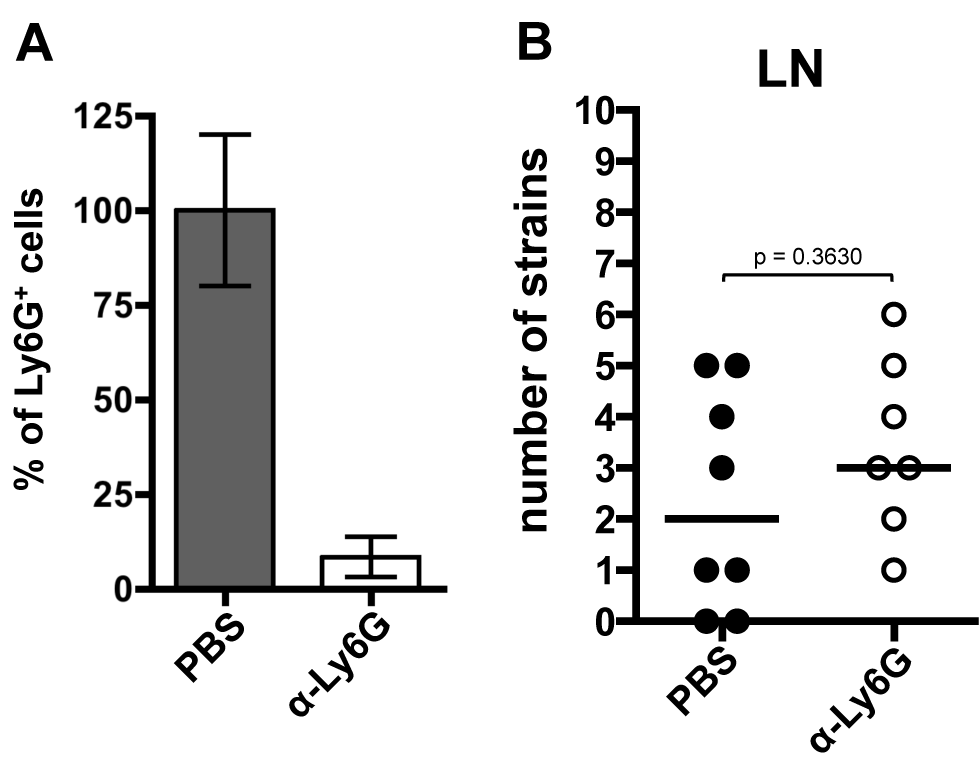

Supplement: S5 Fig — (A) Mean percentage of Ly6G+ cells (neutrophils) in mice injected intravenously (via tail vein) with PBS (gray) or an antibody against Ly6G (white). Data from two combined experiments (3 animals per experiment) are shown. Error bars are standard error of mean (SEM). (B) Number of tagged strains in LNs harvested at 12 hpi after ID inoculation in mice injected with PBS (black) or α-Ly6G (white). The dose used was 189 CFU. Each symbol represents a value from an individual mouse. The horizontal bars indicate medians per group. The Mann Whitney test was used to determine statistical significance. Differences between groups were considered to be statistically significant when p < 0.05. Data from a representative experiment is shown. (TIF) [file ppat.1004587.s005.tif]

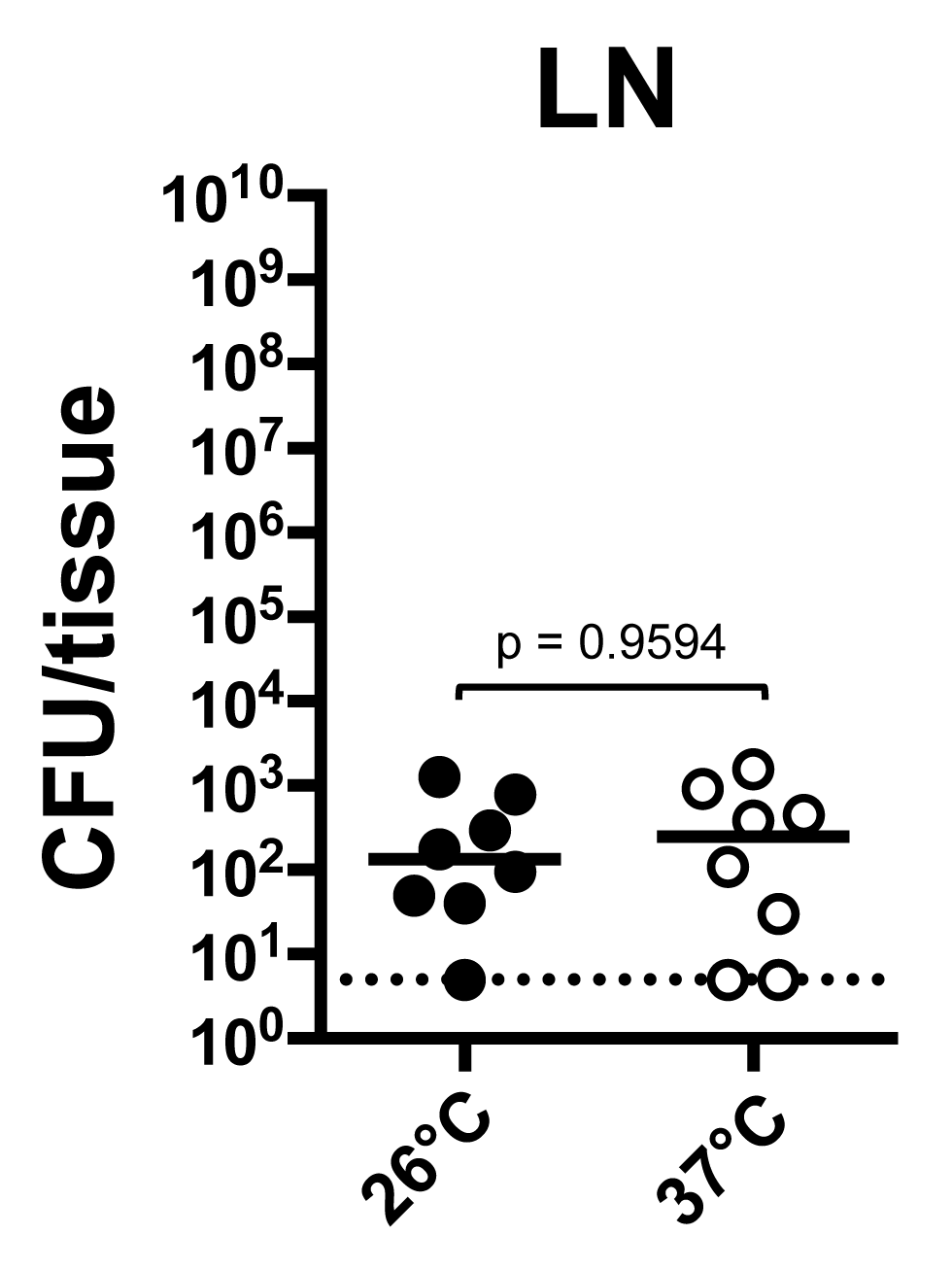

Supplement: S6 Fig — Bacterial burden in LN harvested at 12 hpi from mice inoculated with bacteria grown at 26°C (black) or 37°C (white). A dose of 294 CFU was used for the bacteria grown at 26°C and 269 CFU for the bacteria grown at 37°C. Each symbol represents bacterial burden from a single mouse. Horizontal bars are the median of the group. The dotted line represents the limit of detection. Statistical significance was determined using the Mann Whitney test and the p value is shown. Differences between groups were considered to be statistically significant when p < 0.05. Exposure to bacteria grown at 26°C and 37°C were conducted separately and a single time. (TIF) [file ppat.1004587.s006.tif]

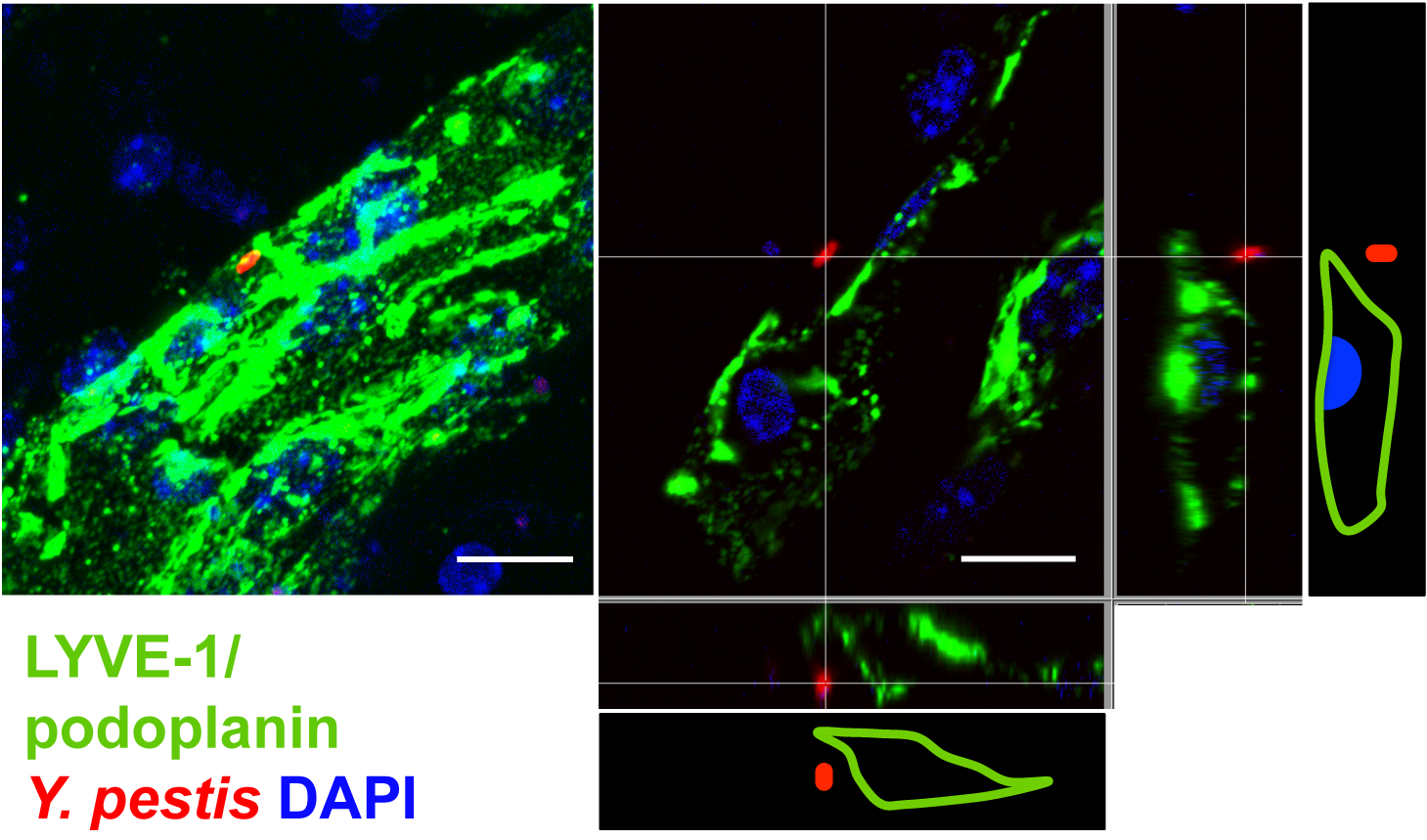

Supplement: S7 Fig — Bacterium (red) in close proximity to a lymphatic vessel (green) 10 min post inoculation. No blue signal (DAPI) is detected near the bacterium. Maximum intensity projection (left) and xz slice (right). Scale bar is 10 μm. Experiments were performed a minimum of 2 times and data from representative experiments are shown. RFP-Y. pestis were used to image bacteria; DAPI was used to identify host cell nuclei; α-LYVE-1 was used to image lymphatic vessels. (TIF) [file ppat.1004587.s007.tif]
