# Supplementary material for: Dissemination of a Highly Virulent Pathogen: Tracking The Early Events That Define Infection
Source: PLoS Pathog. 2015 Jan 22;11(1):e1004587. doi: 10.1371/journal.ppat.1004587 (PMC4303270; doi:10.1371/journal.ppat.1004587)
Supplement: S2 Table — The median frequency of a tagged strain in ears was compared to the equivalent median of each of the other tagged strains. Data from 8 independent experiments were used in the analysis. The table shows the p values obtained after using the Mann-Whitney statistical test to determine whether or not differences between medians were significant. Differences between medians were determined as significant when p < 0.05. Note that only differences between A6 (negative control) and any of the other tagged strains are significant. (PDF) [file ppat.1004587.s009.pdf]

ears	A2	A3	A4	A5	B1	B2	B3	B4	A6
A1	0.2558	0.3745	0.8873	0.7778	0.9845	> 0.999	0.8912	0.8362	0.0002
A2		> 0.999	0.5465	0.6325	0.5445	0.5338	0.3692	0.4906	0.0002
A3			0.5646	0.6068	0.5952	0.5392	0.5377	0.3748	0.0002
A4				0.9851	0.9633	0.9431	0.8695	0.5475	0.0002
A5					> 0.999	0.9329	> 0.999	0.6895	0.0002
B1						> 0.999	0.8990	0.6135	0.0002
B2							0.8679	0.6660	0.0002
B3								0.6131	0.0002
B4									0.0002
